# Probe-based confocal laser endomicroscopy for prediction of uterine rejection in a patient submitted to live-donor uterus transplantation

**DOI:** 10.1590/0102-67202026000008e1937

**Published:** 2026-06-22

**Authors:** Adriana Vaz SAFATLE-RIBEIRO, Dani EJZENBERG, Fernanda Carvalho FRANCO, José Maria SOARES, Edmundo Chada BARACAT, Pedro Augusto MONTELEONE, Wellington ANDRAUS

**Affiliations:** 1 Universidade de São Paulo, Faculty of Medicine, Department of Gastroenterology – São Paulo (SP), Brazil.; 2 Universidade de São Paulo, Instituto do Câncer do Estado de São Paulo, Endoscopy Unit – São Paulo (SP), Brazil.

**Keywords:** Infertility, Transplantation, Uterus, Endoscopy, Microscopy, Fluorescence, Infertilidade, Transplante, Útero, Endoscopia, Microscopia, Fluorescência

## Abstract

Uterus transplantation is a relatively new procedure, with successful births performed using living donors in Sweden since 2014 and a deceased donor, for the first time in Brazil, in 2016. Probe-based confocal endomicroscopy is considered an optical biopsy method (with 1000 times magnification), allowing detailed visualization of tissue cytoarchitecture and microvascular patterns at a penetration depth of approximately 50 to 60 μm. The application of confocal endomicroscopy to the uterine cervix emerges as a promising alternative to weekly cervical examinations in the follow-up of patients who have undergone uterus transplantation. The authors report the case of a 34-year-old woman with Mayer-Rokitansky-Küster-Hauser (MRKH) syndrome, diagnosed at 15 years of age, who in August 2026 underwent the first successful live-donor uterus transplantation performed in Latin America. The surgical procedure was uneventful. The confocal endomicroscopy of the uterine cervix was employed to evaluate its potential utility in identifying inflammatory changes that might precede graft rejection. No irregular or distorted epithelium or severe inflammation was observed, and this finding was confirmed by biopsies and histological analysis. They concluded that the probe-based confocal endomicroscopy may support more effective and individualized post-transplant management, representing a meaningful advancement in the fields of regenerative medicine and transplantation.

## ARTICLE HIGHLIGHTS

Uterus transplantation is a relatively new procedure, with successful births performed using living donors.

More than 100 transplants have already been performed in recent years in various countries around the world, achieving excellent results.

Probe-based confocal endomicroscopy (pCLE) is considered an optical biopsy method (with 1000 times magnification), allowing detailed visualization of tissue cytoarchitecture and microvascular patterns at a penetration depth of approximately 50 to 60 μm.

The application of confocal endomicroscopy to the uterine cervix has previously been reported as an adjunctive tool for guiding targeted biopsies during colposcopy evaluation.

The use of pCLE in the follow-up of patients undergoing uterus transplantation enables early detection of graft rejection while avoiding the risks associated with biopsy.

## Summary

**CENTRAL MESSAGE:**

Uterus transplantation is a relatively new procedure, with successful births performed using living donors in Sweden since 2014 and a deceased donor, for the first time in Brazil, in 2016. Probe-based confocal endomicroscopy (pCLE) is considered an optical biopsy method (with 1000 times magnification), allowing detailed visualization of tissue cytoarchitecture and microvascular patterns at a penetration depth of approximately 50 to 60 μm. The application of confocal endomicroscopy to the uterine cervix emerges as a promising alternative to weekly cervical examinations in the follow-up of patients who have undergone uterus transplantation.

**PERSPECTIVES:**

The use of pCLE in the follow-up of patients undergoing uterus transplantation appears to be a feasible and promising strategy for cervical monitoring, enabling early detection of graft rejection without the risks associated with biopsy. Furthermore, pCLE may support more effective and individualized post-transplant management, representing a meaningful advancement in the fields of regenerative medicine and transplantation.

## RESUMO

O transplante de útero é um procedimento relativamente novo, com nascimentos bem-sucedidos realizados com doadoras vivas na Suécia, desde 2014 e com uma doadora falecida, pela primeira vez no Brasil, em 2016. A endomicroscopia confocal baseada em sonda é considerada um método de biópsia óptica (com ampliação de 1000 vezes) que permite a visualização detalhada da citoarquitetura tecidual e dos padrões microvasculares em uma profundidade de penetração de aproximadamente 50 a 60 μm. A aplicação da endomicroscopia confocal ao colo do útero surge como uma alternativa promissora aos exames cervicais semanais no acompanhamento de pacientes submetidas a transplante de útero. Os autores relatam o caso de uma mulher de 34 anos com síndrome de Mayer-Rokitansky-Küster-Hauser (MRKH), diagnosticada aos 15 anos de idade, que em agosto de 2026 foi submetida ao primeiro transplante de útero bem-sucedido, com doadora viva, realizado na América Latina. O procedimento cirúrgico transcorreu sem intercorrências. A endomicroscopia confocal do colo do útero foi empregada para avaliar sua potencial utilidade na identificação de alterações inflamatórias que possam preceder a rejeição do enxerto. Não foram observadas irregularidades ou distorções no epitélio, nem inflamação grave, e esse achado foi confirmado por biópsias e análises histológicas. Os autores concluíram que a endomicroscopia confocal baseada em sonda pode auxiliar em um manejo pós-transplante mais eficaz e individualizado, representando um avanço significativo nos campos da medicina regenerativa e da transplantação.

## INTRODUCTION

 Uterus transplantation is a relatively new procedure, with successful births performed using living donors in Sweden since 2014^2^ and a deceased donor, for the first time in Brazil, in 2016^4^. It has grown in recent years, with more than 100 transplants already performed in various countries around the world, achieving excellent results^1^. 

 Unlike other solid organs, such as the liver, in which serum enzymes can be markers of organ rejection, the uterus does not have a serum marker that can signal it. This control is usually performed with serial biopsies of the transplanted cervix, which is an invasive examination. 

 Probe-based confocal endomicroscopy (pCLE) is a real-time, *in vivo*, high-resolution imaging technique that has improved the diagnosis of early neoplastic and pre-neoplastic lesions, particularly within the gastrointestinal tract^6^. It is considered an optical biopsy method (with 1000 times magnification), allowing detailed visualization of tissue cytoarchitecture and microvascular patterns at a penetration depth of approximately 50 to 60 μm. 

 The application of confocal endomicroscopy to the uterine cervix has previously been reported as an adjunctive tool for guiding targeted biopsies during colposcopy evaluation^3,6,7^. Given that the cervical epithelium typically measures between 50 and 100 μm in thickness, pCLE emerges as a promising alternative to weekly cervical examinations in the follow-up of patients who have undergone uterus transplantation. 

## CASE REPORT

 We report the case of a 34-year-old woman with MayerRokitansky-Küster-Hauser (MRKH) syndrome, diagnosed at 15 years of age. MRKH syndrome is a congenital anomaly resulting from failure of Müllerian duct development, leading to uterine agenesis and partial or complete vaginal absence. Affected individuals typically present with normal female external genitalia and normal secondary sexual characteristics, including breast development and pubic hair. 

 In August 2024, after approval from an Ethics Committee of the institution (number 81297224.8.0000.0068) and written informed consent, the patient underwent the first successful live-donor uterus transplantation performed in Latin America. The surgical procedure was uneventful. Postoperatively, the patient was maintained on immunosuppressive therapy with tacrolimus, azathioprine and prednisone. Weekly cervical biopsies were performed to detect early signs of graft rejection. 

 pCLE of the uterine cervix was performed to evaluate its potential utility in identifying inflammatory changes that might precede graft rejection. Two sessions of pCLE were conducted on postoperative days 10 and 52. Both procedures were carried out without sedation, with the patient in lithotomy position, following intravenous administration of fluorescein. 

**Figure 1:**
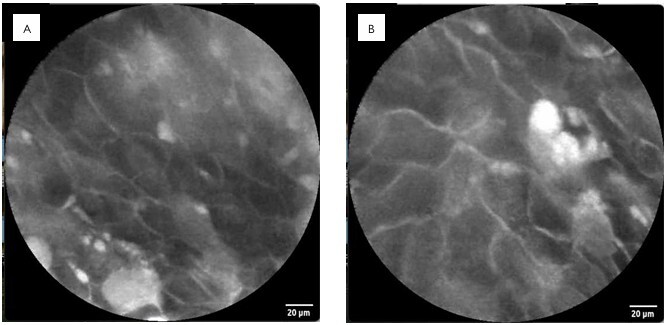
(A and B). pCLE shows regular squamous cells, normal intra-papillary capillary loops, and scattered inflammatory cells.

**Figure 2:**
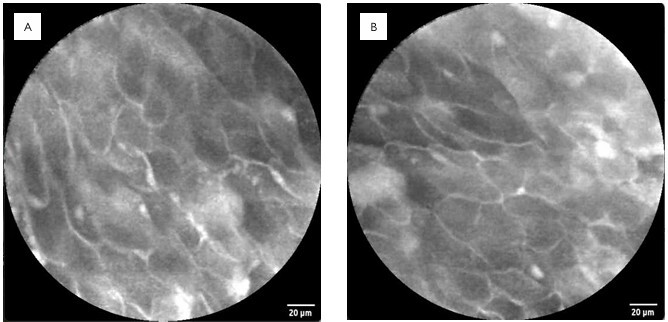
(A and B). pCLE shows regular squamous cells, slightly fluorescein extravasation between them, and no features of rejection.

 The first pCLE cervix examination demonstrated preserved squamous epithelium with regular squamous cells, normal intrapapillary capillary loops, and scattered inflammatory cells (Figures 1A and 1B). The second pCLE examination revealed regular squamous cells, slight fluorescein extravasation between them, and no features of rejection. No irregular or distorted epithelium or severe inflammation was observed (Figures 2A and 2B). 

**Figure 3:**
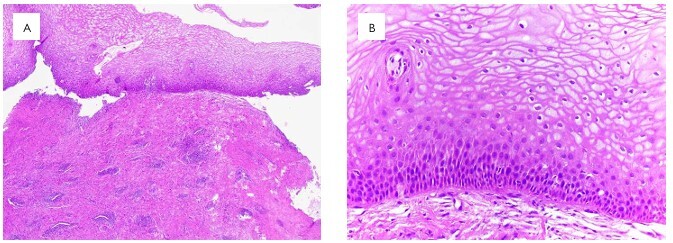
(A and B). Minimal spongiosis and scant intraepithelial lymphocytes are present. No evidence of rejection (H&E, 40X and 200X).

**Figure 4:**
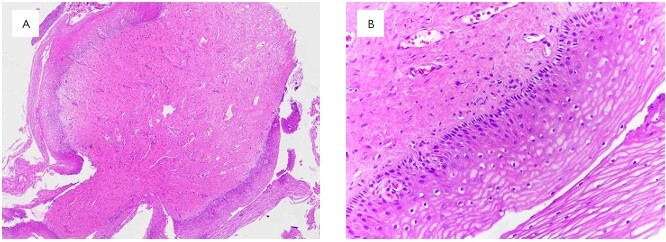
(A and B). Ectocervix showing minimal spongiosis and no evidence of rejection (H&E, 40X and 200X).

 To assess the diagnostic accuracy of pCLE and to confirm the histological findings, targeted cervical biopsies were obtained immediately following each procedure. Histological analysis confirmed no signs of rejection in both instances. At the first evaluation, histology demonstrated minimal spongiosis and sparce intraepithelial lymphocytes (Figures 3A and 3B). At the second evaluation, histological findings were limited to spongiosis (Figures 4A and 4B). 

 A few months later, she underwent *in vitro* fertilization and became pregnant. The pregnancy was uneventful. 

## DISCUSSION

 Uterus transplantation has been growing worldwide, and the number of transplant recipients and births through this method has increased over the years, with prospects for continued growth in the future^5^. Because it is a relatively new transplant method, the assessment of organ rejection is not yet fully established in global experience. Furthermore, it lacks a reliable serum marker. 

 pCLE enables detailed analysis of the cervical tissue cytoarchitecture and local vascularization. Similarly to histology, it may allow early detection of inflammatory cells and features suggestive of graft rejection, while mitigating the risks associated with repeated biopsies in an immunosuppressed patient. 

 Through *in vivo* examination of the cervix, this minimally invasive approach has the potential to decrease the need for frequent invasive procedures, thereby minimizing procedurerelated complications, including bleeding and post-procedural infections, and providing a safer surveillance strategy. Moreover, pCLE may reduce costs by limiting unnecessary biopsies and subsequent histopathological analyses. 

## CONCLUSIONS

 The use of pCLE in the follow-up of patients undergoing uterus transplantation appears to be a feasible and promising strategy for cervical monitoring, enabling early detection of graft rejection while avoiding the risks associated with biopsy. Furthermore, pCLE may support more effective and individualized post-transplant management, representing a meaningful advancement in the fields of regenerative medicine and transplantation. 

## Data Availability

The datasets generated and/or analyzed during the current study are available from the corresponding author upon reasonable request.
